# Indocyanine green lymphangiography is superior to clinical staging in breast cancer-related lymphedema

**DOI:** 10.1038/s41598-021-00396-2

**Published:** 2021-10-26

**Authors:** Mads Gustaf Jørgensen, Anne Pernille Hermann, Anette Riis Madsen, Steffanie Christensen, Jens Ahm Sørensen

**Affiliations:** 1 Department of Plastic and Reconstructive Surgery, Odense University Hospital, J. B. Winsløws Vej 4, 5000 Odense, Denmark; 2 Research Unit for Plastic Surgery, University of Southern Denmark, Odense, Denmark; 3 OPEN, Open Patient Data Explorative Network, Odense University Hospital, Odense, Denmark; 4 Department of Endocrinology, Odense University Hospital, Odense, Denmark

**Keywords:** Breast cancer, Diagnosis, Physical examination

## Abstract

Precise staging of breast cancer-related lymphedema (BCRL) is important to guide treatment-decision making. Recent studies have suggested staging of BCRL using indocyanine green lymphangiography (ICG-L) based on the extent of lymphatic injury and dermal backflow patterns. Currently, the benefits of ICG-L compared to conventional clinical staging are unknown. For this study, we included 200 patients with unilateral BCRL. All BCRL patients were staged using ICG-L and clinical exam. The amounts of excess arm volume, fat mass and lean mass were compared between stages using Dual Energy X-Ray Absorptiometry. Multivariate regression models were used to adjust for confounders. For each increase in the patient's ICG-L stage, the excess arm volume, fat mass and lean mass was increased by 8, 12 and 6.5 percentage points respectively (*P* < 0.001). For each increase in the patient's clinical ISL stage, the volume was increased by 3.5 percentage points (*P* < 0.05), however no statistically significant difference in the lean and fat mass content of the arm was observed for ascending stages. However, the residual plots showed a high degree of variance for both ICG-L and clinical staging. This study found that ICG-L staging of BCRL was superior to clinical staging in forecasting BCRL excess arm volume, fat mass, and lean mass. However, there was a high degree of variance in excess arm volume, fat mass, and lean mass within each staging system, and neither the ICG-L nor clinical staging forecasted perfectly.

## Introduction

The heart of breast cancer-related lymphedema (BCRL) is the lymphatic injury after lymphadenectomy and radiotherapy^1,2^. Traditionally, BCRL has only been assessed clinically^3^, but the disadvantage of clinical evaluation is that it is subjective, user-dependent and fails to address the underlying lymphatic injury^4^. Indocyanine green lymphangiography (ICG-L) has received considerable attention over the last decade, as it allows bedside identification of lymphatic vessels and flow in real-time^5^. Previous research has established a disease staging system of BCRL using ICG-L^6,7^, based on the visual severity of the lymphatic injury and lymphatic dermal backflow patterns. Currently, the benefits of ICG-L over clinical staging have not been established, and much less is known whether the degree of lymphatic injury is associated with BCRL severity^3^.

Therefore, this study aimed to investigate the usefulness of ICG-L staging by comparing ICG-L and clinical staging of BCRL to the excess arm volume, fat, and lean mass. We hypothesized that ICG-L would be more appropriate than clinical staging at stratifying BCRL patients.

## Methods

### Participants

This cross-sectional study included BCRL patients referred to our clinic for experimental lymphedema treatment between January 2019 and February 2020. All patients were screened for study eligibility per email and telephone and were explicitly invited for participation in this study based on the following criteria:

- Unilateral arm lymphedema diagnosed by a lymphedema physiotherapist
- Previous treatment for loco-regional breast cancer with axillary lymph node dissection
- Cancer-free for more than one year
- Lymphedema for more than one year and currently in stable lymphedema treatment
- Body mass index ≤ 35
- ASA 1 or 2
- Able to communicate in Danish
- No history of other malignancy apart from breast cancer and non-melanoma skin cancer
- No insulin-dependent diabetes
- No known hepatitis, HIV, or syphilis infection
- No primary lymphedema or non-breast cancer-related lymphedema
- Severe thyroid disease and known indocyanine-green allergy (contraindication for ICG-L)

All included patients signed informed consent and underwent Dual Energy X-Ray Absorptiometry scans, clinical evaluation, and ICG-L in that order.

In addition, we registered the following demographic information for each patient: Age (years), marital status (yes/no), employment status (yes/no), time of lymphedema diagnosis, previous arm cellulitis since lymphedema diagnosis (yes/no), arm laterality of lymphedema (right/left), arm dominance (right/left) and use of conservative lymphedema treatment (compression sleeve (yes/no), gauntlet(yes/no), night compression(yes/no) and pneumatic compression devices(yes/no). The patient's current weight and height were measured in the outpatient clinic, and the body mass index was calculated. The following information regarding previous breast cancer treatment were retrieved from the Danish Breast Cancer Group registry^8^: type of breast surgery(mastectomy/lumpectomy), radiation therapy(yes/no), chemotherapy(yes/no), and the number of lymph nodes removed in axillary dissection.

### Estimation of excess arm volume, fat, and lean mass

Dual Energy X-Ray Absorptiometry (DEXA) was used to estimate the excess arm volume, fat, and lean mass in the BCRL arm compared to the healthy contralateral arm. DEXA is a three-compartment scan modality that measures bone mass, fat mass, and lean mass (e.g., muscle, fluid, and fibrosis). All DEXA scans were performed using the Discovery/Horizon A densitometer (Hologic, Waltham, Massachusetts, USA, serial number 82245/301872 M) and analyzed using the Hologic APEX software version 13.3:3/13.6.0.5:3. All participants were positioned supine on the scan table with their heads at the top of the scan table. To capture both the lymphedema and healthy arms, we performed two whole-body DEXA scans on each patient, which were analyzed using sub-regions. Patients were shifted laterally from the table's midline, with the arms extended straight at a slight angle to not overlap with the torso and thighs. The patient's hands were positioned palmar side down with separated fingers. Patients were further instructed to dress lightly, remove all metal items, jewelry or watches before each scan. All scans were performed during the daytime. Region of interests were manually drawn for each arm extending from the tip of the finger and 25 software-standardized units proximally. Assessments of DEXA scans were performed by blinded assessors, which were not involved with the clinical staging or ICG-L staging. For both arm regions, the amount of fat mass, lean mass, and bone mass was retrieved in grams and the volume calculated using known densities^9,10^.

### Clinical staging of BCRL

The gold standard conventional International Society of Lymphology (ISL) staging system was used to stage BCRL patients clinically:

- Stage 0: No swelling, subtle physical changes, and subjective BCRL symptoms.
- Stage 1: Mild swelling that subsides with limb elevation and compression.
- Stage 2a: Early moderate swelling with pitting-edema that does not improve significantly with elevation or compression.
- Stage 2b: Late moderate swelling without pitting-edema that does not significantly improve limb elevation or compression.
- Stage 3: Severe swelling with significant trophic skin thickness.

The clinical staging was performed blinded by persons not involved with the assessments of DEXA scans or assessments of ICG-L grading.

### ICG-L staging of BCRL

The ICG-L was performed and patients were staged using the MD Anderson staging system as previously described^6,7,11^ In brief, we injected 0.1 ml ICG (2.5 mg/ml Verdye, Diagnostic Green, Ascheim, Germany) into the ulnar border of the palmaris longus tendon at the wrist's level, and all scans were performed using the HyperEye medical system (MNIRC-501, HEMS; Mizuho Co., Tokyo, Japan). The entire BCRL arm was scanned approximately 1 h and the scan was saved for subsequent review by a blinded observer. The ICG-L scan recording was evaluated by a blinded observer, which was not involved with the clinical staging or DEXA scans. The MD Anderson ICG-L staging systems comprise of 6-stages, and the degree of lymphatic injury and lymphatic dermal backflow is graded on a 0–5 scale:

- Stage 0: Normal linear lymphatics and no dermal backflow
- Stage 1: Many patent lymphatics and minimal lymphatic dermal backflow
- Stage 2: Moderate number of patent lymphatics and segmental dermal backflow
- Stage 3: Few patent lymphatics and extensive dermal backflow
- Stage 4: Dermal backflow involving the hand
- Stage 5: No proximal uptake of ICG from the injection site

Following ICG injection, we monitored all patients for at least 60 min for allergic and hypersensitive reactions to the dye.

### Statistical methods

We described the baseline characteristics of patients with means ± standard deviation (SD) for continuous parametric variables, median and interquartile range (IQR) for nonparametric continuous variables, and rounded frequencies (%) for categorical variables. The Skewness/Kurtosis test was used to test for normal distributions of continuous variables.

A multivariate linear regression model was used to analyze the excess arm volume, fat, and lean mass associated with incremented ICG-L and ISL stage. A multivariate ordered logistic regression model was used to analyze the association between ICG-L and ISL staging. The model's goodness of fit was tested using the likelihood-ratio test. Clinically relevant variables (age, BMI, arm dominance, lymphedema duration, and lymphedema latency) were a priori included in the multivariate regression models^12^. STATA 15 (StataCorp. 2017. Stata Statistical Software: Release 15. College Station, TX: StataCorp LP) was used for the statistical analysis and conducted with a two-tailed significance level of 0.05 and reported with 95% CI when applicable.

### Ethical approval

This study was registered with the Danish Data Protection Agency (2008‐58‐0035) and approved by The Regional Committees on Health Research Ethics for Southern Denmark (S-20180117). All patients signed informed consent before the assessments. All methods were carried out in accordance with relevant guidelines and regulations.

## Results

**Figure 1:**
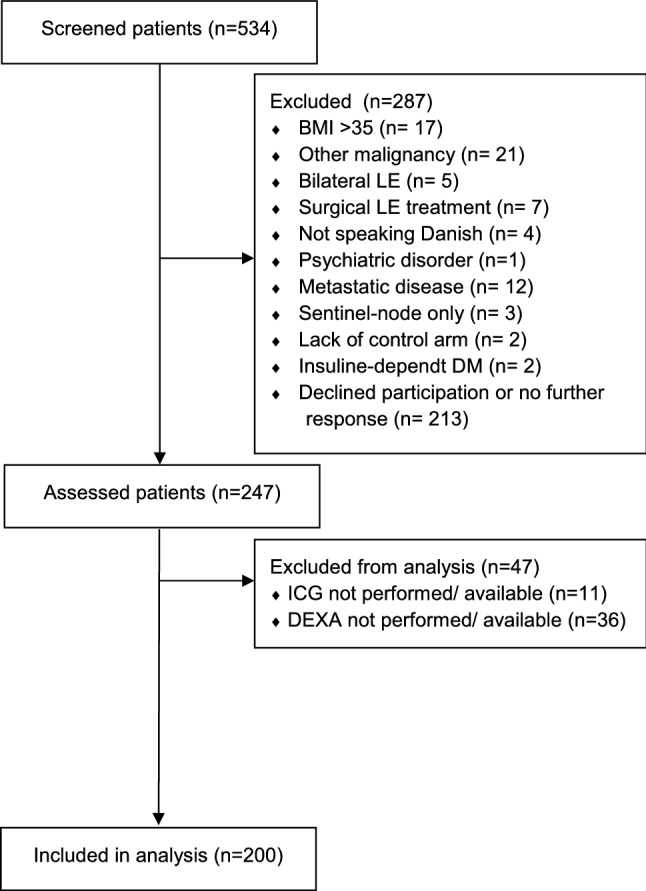
This figure shows the flowchart of the include patients.

We assessed 247 BCRL patients, of which 200 patients were ultimately included between January 2019 and February 2020 (Fig. 1). We excluded 47 patients on-site due to ICG-L being occupied in the operating theatre and malfunctions of the DXA scanner. The baseline demographics of all included patients are summarized in Table 1.

**Table 1:** Demographic and characteristics of included patients.

Variables	Data distribution	All patients (n = 200)
Age (years)	Mean ± SD	59.46 ± 10.05
BMI (kg/m^2^)	Median (IQR)	27.44 (7.27)
In a relationship (yes)	N (%)	140 (70.00%)
Employed (yes)	N (%)	117 (58.50%)
**Breast cancer treatment**		
Radiation therapy (yes)	N (%)	187 (93.50%)
Chemotherapy (yes)	N (%)	167 (83.92%)
Endocrine therapy (yes)	N (%)	158 (80.20%)
Lymph nodes removed (N)	Median (IQR)	17 (8)
Mastectomy (yes)	N (%)	107 (53.50%)
Post-mastectomy reconstruction (yes)	N (%)	48 (44.86%)
*Abdominal free flap (yes)*	N (%)	21 (19.63%)
*Pedicled back flap (yes)*	N (%)	15 (14.02%)
*Implant-based reconstruction (yes)*	N (%)	12 (11.21%)
**Lymphedema characteristics**		
Lymphedema latency (years)	Median (IQR)	0.67 (1.43)
Lymphedema duration (years)	Median (IQR)	4.40 (5.52)
Lymphedema volume (mL)	Mean ± SD	216.98 ± 216.75
Lymphedema volume (%)	Mean ± SD	22.71 ± 22.91
Lymphedema in dominant arm (yes)	N (%)	95 (47.50%)
A previous episode of cellulitis (yes)	N (%)	68 (34.34%)
**Current lymphedema treatment**		
Compression sleeve (yes)	N (%)	175 (87.50%)
Compression gauntlet (yes)	N (%)	122 (61.00%)
Night compression (yes)	N (%)	63 (31.50%)
Pneumatic compression device (yes)	N (%)	39 (19.50%)

This table shows the baseline characteristics of the included patients. N = number, IQR = interquartile range.

*SD* standard deviation.

**Figure 2:**
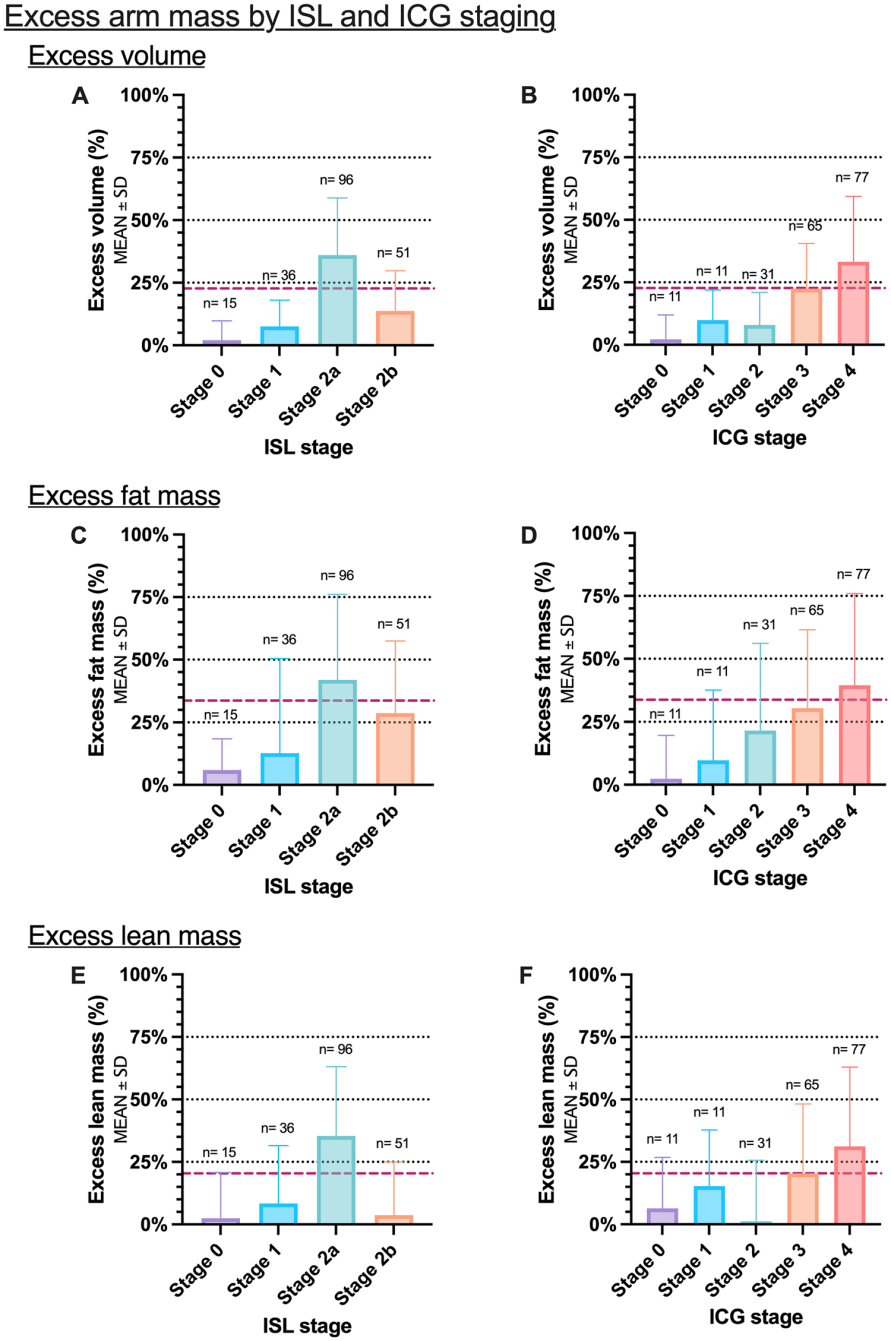
This figure shows the distribution of excess arm volume, fat and lean mass in lymphedema patients stratified by clinical ISL and lymphangiography staging. (**A**) Excess volume and ISL stage. (**B**) Excess volume and ICG stages. (**C**) Excess fat mass and ISL stage (**D**) Excess fat ass and ICG stages. (**E**) Excess lean mass and ISL stage (**F**) Excess lean mass and ICG stages. Black horizontal dotted lines denote 25%, 50%, and 75%. The red striped line denotes the mean excess volume, fat and lean mass for the study. *P-value < 0.05. **P-value < 0.001.

Excess arm volumes increased from ISL stage 0 to stage 1 and 2a but was lower for stage 2b than 2a (Fig. 2A). In contrast, excess arm volumes increased with each incremental ICG stage (Fig. 2B). Similarly, Excess arm fat mass increased from ISL stage 0 to stage 1 and 2a but was again lower in stage 2b than for stage 2a (Fig. 2C). Again, excess fat mass increased incrementally with each ICG stage. The same pattern was seen in excess arm lean mass. Excess arm lean mass increased from ISL stage 0 to stage 1 and 2a but was lower in stage 2b than for stage 2a and stage 1 patients (Fig. 2D). Surprisingly, excess arm lean mass increased from ICG stage 0, 1, 3 and 4–but was lower in stage 2 than all other stages (Fig. 2E).

The multivariate ordinal logistic regression showed that for each increase in the ISL stage, there was only an odds ratio of 1.53 (95%CI 1.22; 1.93, *P* < 0.001) for also having an increasing ICG stage.

The multivariate linear regression showed that for each increase in the patient's clinical ISL stage, the volume was increased by 3.5 percentage points (*P* < 0.05), however no statistically significant difference in the lean and fat mass content of the arm was observed for ascending stages (Table 2). In contrast, for each increase in the patient's ICG-L stage, the volume was increased by 8 percentage points (Table 3), the fat mass by 12 percentage points, and the lean mass content by 6.5 percentage points (*P* < 0.001).

**Table 2:** Clinical lymphedema stage and excess volume, fat mass, and lean mass.

Clinical International Society of Lymphology stage							
Arm composition	Stage 0 (n = 15)	Stage 1 (n = 36)	Stage 2a (n = 96)	Stage 2b (n = 51)	Stage 3 (n = 2)	Multivariate linear regression	
	Mean ± SD	Mean ± SD	Mean ± SD	Mean ± SD	Mean ± SD	Coefficient (95%CI)	*P*-value
Volume (%)	2.00 ± 7.77	7.52 ± 10.51	36.01 ± 22.83	13.71 ± 16.09	42.98 ± 24.52	3.64 (0.09; 7.21)	= 0.04
Fat mass (%)	2.38 ± 17.21	9.72 ± 27.83	21.55 ± 34.58	30.48 ± 31.10	48.38 ± 66.06	7.23 (− 0.48; 14.95)	= 0.07
Lean mass (%)	2.44 ± 18.36	8.31 ± 23.16	35.37 ± 27.77	3.72 ± 21.26	79.57 ± 82.86	2.95 (− 17.61; 23.53)	= 0.77

This table shows the association between clinical lymphedema stage and excess volume, fat mass, and lean mass. The multivariate regression model was adjusted for age, BMI, arm dominance, lymphedema duration, and lymphedema latency. N = number of patients at each stage.

*SD* standard deviation.

**Table 3:** Indocyanine green lymphangiography stage and excess volume, fat mass, and lean mass.

Indocyanine green lymphangiography stage								
Arm composition	Stage 0 (n = 11)	Stage 1 (n = 11)	Stage 2 (n = 31)	Stage 3 (n = 65)	Stage 4 (n = 77)	Stage 5 (n = 5)	Multivariate linear regression	
	Mean ± SD	Mean ± SD	Mean ± SD	Mean ± SD	Mean ± SD	Mean ± SD	Coefficient (95%CI)	*P*-value
Volume (%)	4.38 ± 6.6 6	10.04 ± 6.64	10.16 ± 7.27	21.49 ± 12.88	24.38 ± 14.65	14.34 ± 9.52	8.15 (5.68; 10.63)	< 0.001
Fat mass (%)	2.38 ± 17.21	9.72 ± 27.83	21.55 ± 34.58	30.48 ± 31.10	48.38 ± 66.06	49.79 ± 34.27	11.96 (6.28; 17.63)	< 0.001
Lean mass (%)	6.37 ± 20.41	15.30 ± 22.48	1.23 ± 24.39	20.38 ± 27.86	31.14 ± 31.69	16.15 ± 27.91	6.56 (3.15; 9.98)	< 0.001

This table shows the association between lymphangiography lymphedema stage and excess volume, fat mass, and lean mass. The multivariate regression model was adjusted for age, BMI, arm dominance, lymphedema duration, and lymphedema latency. N = number of patients at each stage.

*SD* standard deviation.

**Figure 3:**
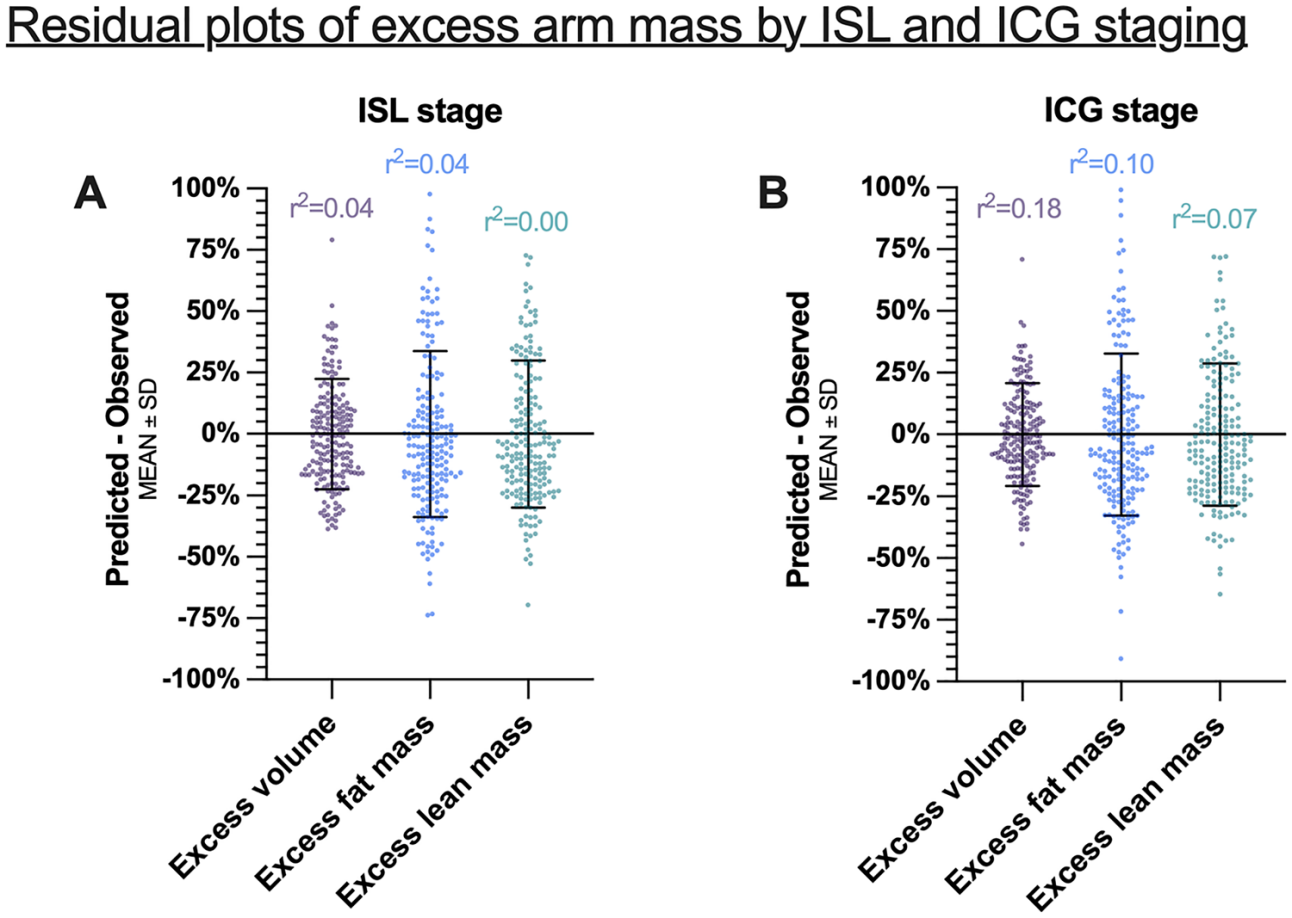
This figure shows the residuals of predicted minus observed excess volume, fat, and lean mass by (**A**) ISL stage and (**B**) ICG stage.

The residual plot analysis of the regression models showed that there was a high degree of variance regarding excess arm volume, fat mass, and lean mass in each ISL (Fig. 3A) and ICG stage (Fig. 3B).

There was an expected overlap between the ICG and ISL staging systems (Table 4). Half of the patients with ICG stage 0 (5/11, 45.45%) were also staged as ISL stage 0. The majority of patients that were ICG stage 1 (6/11, 54.55%) were also ISL stage 1. Surprisingly, the majority of patients that were ICG stage 2 (14/31, 45.16%) were staged as ISL stage 2b, while most patients that were ICG stage 3 (37/65, 56.92%) and 4 (52/77, 67.53%) was ISL stage 2a.

**Table 4:** Indocyanine green lymphangiography and clinical lymphedema staging contingency.

		Indocyanine Green Lymphangiography stage											
		0 (n = 11)		1 (n = 11)		2 (n = 31)		3 (n = 65)		4 (n = 77)		5 (n = 5)	
International Society of Lymphology stage	0 (n = 15)	5	33.33%	1	6.67%	6	40.00%	1	6.67%	1	6.67%	1	6.67%
		45.45%		9.09%		19.35%		1.69%		1.30%		20.00%	
	1 (n = 36)	4	11.11%	6	16.67%	7	19.44%	9	25.00%	10	27.78	0	0.00%
		36.36%		54.55%		22.58%		13.85%		12.99		0.00%	
	2a (n = 96)	0	0.00%	1	1.04%	4	4.16%	37	38.54%	52	54.17%	2	2.08%
		0.00%		9.09%		12.90%		56.92%		67.53%		40.00%	
	2b (n = 51)	2	3.92%	3	5.88%	14	27.45%	18	35.29%	12	23.53	2	2.08%
		18.18%		27.27%		45.16%		27.69%		15.58%		40%	
	3 (n = 2)	0	0.00%	0	0.00%	0	0.00%	0	0.00%	2	100%	0	0.00%
		0.00%		0.00%		0.00%		0.00%		2.60%		0.00%	

This table shows the contingency between the Indocyanine Green Lymphangiography stage and the International Society of Lymphology stage. N = number of patients at each stage.

## Discussion

This study found that lymphangiography staging using ICG-L was superior to clinical staging using the ISL standards in forecasting BCRL excess arm volume, fat mass, and lean mass. However, there was a high degree of variance in excess arm volume, fat mass, and lean mass within each staging system, and neither the ICG-L nor ISL staging systems forecasted perfectly.

BCRL is most often diagnosed and staged clinically due to its immediate accessibility, speed, and low cost. In contrast, ICG-L is time-consuming, has a high entry cost (some costing more than 50.000$) and carries a small risk of allergic adverse reactions, which can limit its broad implementation. Furthermore, medical attendance is often required for ICG-L due to the applied methods and risk of allergic adverse events. Before routine implementation of ICG-L for the diagnosis and staging for BCRL, there is a need for restructuring the setting for BCRL assessments. The main limitation of this study and ICG-L staging in general is that ICG-L only provides a two-dimensional snapshot of the lymphatic system with a limited depth of 2 cm below the skin. However, despite this technical limitation, superficial ICG-L evaluation seems to reflect the overall condition of the limb well. In contrast to ICG-L, lymphoscintigraphy and magnetic resonance are imaging modalities that can visualize the superficial and also the deep lymphatic system^13^. However, the advantage of ICG-L over lymphoscintigraphy and magnetic resonance imaging is its bedside usability, lower cost, non-radioactive contrast agent, and high resolution enabling real-time identification of static and dynamic lymphatic vessels and flows^14^. Currently, there is no universally accepted method for BCRL assessments, as all imaging modalities have their pros and cons. Future comparative studies are needed to determine the best imaging modality or combination for accurate evaluation of BCRL^15^. For example using a dual-imaging approach, combining both ICG-L and magnetic resonance imaging or photoacoustic lymphangiography to capture both the superficial and deep lymphatic system^16,17^. Nevertheless, this study also has several strengths, such as its large sample size with complete demographic information and regression models to adjust for known confounders. Another major strength of this study was the blinding of clinical, lymphangiography, and DEXA assessments which should minimize the risk of observation and confirmation bias.

Patients with BCRL have significantly impaired quality of life^18^. To improve the burden of BCRL it is important to adequate stage the disease and guide the most optimal treatment path. This study showed that lymphangiography staging of BCRL was superior to clinical staging, which may have implications for guiding microsurgical and conservative treatment decision-making. However, only a few previous studies have previously compared lymphatic imaging to clinical findings. Thomis et al. investigated the correlation between ICG-L stage and pitting, skinfold thickness and skin elasticity and found a weak to moderate correlation^19^. Their finding was supported by Garza et al., which showed a poor correlation between ICG-L and ISL stage and that ISL stage alone was insufficient in selecting BCRL patients for microsurgical treatments at the authors institution^20^. One reason for this may be due to the large degree of variance in excess arm volume, fat mass and lean mass for both clinical and lymphangiography staging found in our study. Such large variances in excess arm masses can make stratification of patients difficult and imprecise. Nevertheless, our study found that the variance was slightly better using ICG-L compared to ISL staging. Additionally, ICG-L was better at modelling the amount of excess volume, fat mass and lean mass than ISL staging. This suggest that ICG-L staging may be more suitable in patient-stratification than ISL staging. There are several additional benefits of ICG-L evaluation of BCRL beyond patient stratification. Bedside visualization of the lymphatic injury’s location and severity can empower the patients understanding of their condition and treatment rationale^21^. Additionally, ICG-L can reveal intact sternal, clavicular, and axillary drainage pathways to guide personalized microsurgical treatments and conservative treatment^22,23^.

The contingency between clinical and lymphangiography staging showed an interesting overlap between ISL and ICG-L staging. The contingency analysis showed that more clinically stage 2a patients were actually staged higher on ICG-L than clinical stage 2b patients. Furthermore, stage 2a patients had more excess lean mass than stage 2b patients, but a comparable amount of excess fat mass. These interesting findings may be interpreted as stage 2a patients actually having more severe lymphatic injury than stage 2b patients. This is surprising, as clinically stage 2b have traditionally been perceived as the “worse” clinical stage, due to the belief that the fluid portion of lymphedema in stage 2a have been converted to fibrofatty tissue in stage 2b^24^. In contrast to this notion, the current study offers compelling evidence that clinical stage 2a patients have more severe uncompensated or “active” lymphedema than stage 2b patients. However the reason for clinical stage 2a patients having more severe lymphatic injury can only be speculated upon. One possible explanation may be due to the overlapping clinical criteria of significant pitting-edema in stage 2a, which seems to be correlated to a more severe degree of lymphatic injury and leakage as visualized on ICG-L. Future studies are clearly needed to understand why some patients develop severe arm lymphatic injury and others no or mild lymphatic injury after breast cancer treatment.

Prominent BCRL treatments such as microsurgery and conservative physiotherapy aim at reducing the excess fluid in the arm and the indication for treatment is often made clinically. However, our results show that clinical stage 0, 1, and 2b patients have very little excess fluid-like mass compared to stage 2a. This may explain the inconsistent treatment results observed after microsurgical and physiotherapeutic lymphedema treatments^25–29^. Regardless, future studies are needed to elucidate whether the excess amounts of volume, fat and lean mass can predict which patients are more likely to benefit from selective BCRL treatments^30,31^.

## Conclusion

This study found that lymphography staging using ICG-L was superior to clinical staging alone for stratifying BCRL patients. ICG-L staging may have implications when treating BCRL, whether it being surgical or conservative.

## Data Availability

The study data files are housed on institutional storage and are not publicly available for the following reason: data contain information that could compromise research participant privacy. However, the data can be made available upon reasonable request to the corresponding author in accordance with the institutional data sharing policy as part of an external collaborative request. The individual-level data are not publicly available because of data privacy regulations and restrictions for use of such data, as stated in the study protocol and patient consent form.
